# Epigenetic Regulation of Host Defense Peptide Synthesis: Synergy Between Histone Deacetylase Inhibitors and DNA/Histone Methyltransferase Inhibitors

**DOI:** 10.3389/fimmu.2022.874706

**Published:** 2022-04-22

**Authors:** Melanie A. Whitmore, Hong Li, Wentao Lyu, Sharmily Khanam, Guolong Zhang

**Affiliations:** ^1^ Department of Animal and Food Sciences, Oklahoma State University, Stillwater, OK, United States; ^2^ College of Animal Science and Technology, Henan Agricultural University, Zhengzhou, China; ^3^ State Key Laboratory for Managing Biotic and Chemical Threats to the Quality and Safety of Agro-Products, Institute of Agro-Product Safety and Nutrition, Zhejiang Academy of Agricultural Sciences, Hangzhou, China; ^4^ National Center for Toxicological Research, Food and Drug Administration, Jefferson, AR, United States

**Keywords:** host defense peptides, histone methyltransferase inhibitors, DNA methyltransferase inhibitors, histone deacetylase inhibitors, epigenetic modification

## Abstract

Host defense peptides (HDPs) are an integral part of the innate immune system acting as the first line of defense. Modulation of HDP synthesis has emerged as a promising host-directed approach to fight against infections. Inhibition of histone deacetylation or DNA methylation is known to enhance HDP gene expression. In this study, we explored a possible synergy in HDP gene induction between histone deacetylase inhibitors (HDACi) and DNA/histone methyltransferase inhibitors (DNMTi/HMTi). Two chicken macrophage cell lines were treated with structurally distinct HDACi, HMTi, or DNMTi individually or in combinations, followed by HDP gene expression analysis. Each epigenetic compound was found to be capable of inducing HDP expression. To our surprise, a combination of HDACi and HMTi or HDACi and DNMTi showed a strong synergy to induce the expressions of most HDP genes. The HDP-inducing synergy between butyrate, an HDACi, and BIX01294, an HMTi, were further verified in chicken peripheral blood mononuclear cells. Furthermore, tight junction proteins such as claudin 1 were also synergistically induced by HDACi and HMTi. Overall, we conclude that HDP genes are regulated by epigenetic modifications. Strategies to increase histone acetylation while reducing DNA or histone methylation exert a synergistic effect on HDP induction and, therefore, have potential for the control and prevention of infectious diseases.

## Introduction

Antimicrobial resistance has become a global health crisis due to misuse and overuse of antimicrobials in both human and food animals (1, 2). Host defense peptides (HDPs) are an essential component of the innate immune system providing the first line of defense against bacterial, viral, fungal, and parasitic infections in almost all living organisms (3, 4). Two major families of HDPs, known as defensins and cathelicidins, exist in vertebrate animals (3, 4). There are a total of 14 β-defensins known as AvBD1-14 and four cathelicidins known as CATH1-3 and CATHB1 in chickens (5, 6). With a net positive charge and amphipathic structure, these small peptides kill pathogens primarily through electrostatic interactions and subsequent disruption and rupture of membranes (3, 4). HDPs have also been found to actively participate in chemotaxis, endotoxin neutralization, wound healing, and maintenance of barrier integrity (7, 8). Hence, employing HDPs against infections has emerged as a novel host-directed therapeutic approach (3, 4, 8, 9). Specifically, modulation of HDP synthesis is being actively explored as an antibiotic-alternative strategy for disease control and prevention (9–11).

Epigenetic modifications of histones or DNA have been found to profoundly impact gene transcription (12, 13). Histones are covalently modified in a variety of ways such as methylation and acetylation, while DNA is modified by methylation and demethylation (12, 13). Acetylation of histones occurs through histone acetyltransferases (HAT) and deacetylation through histone deacetylases (HDAC). Histones are also methylated by a group of histone methyltransferases (HMT) and demethylated by histone demethylases, while DNA is methylated by DNA methyltransferases (DNMT) and demethylated actively by various enzymes or passively during DNA replication (14). Hyperacetylation of histones as well as hypomethylation of DNA or histones is generally associated with active gene transcription, while histone hypoacetylation or DNA/histone hypermethylation often leads to transcription suppression (12, 13). HDAC inhibitors (HDACi) are classified into hydroximates, cyclic peptides, aliphatic acids, and benzamides based on their structural differences (15). A number of HDACi such as butyrate are well known to induce HDP gene expression (10, 16), suggesting a positive role of histone acetylation in HDP regulation. Several HDP genes have been found to be upregulated by DNMT inhibitors (DNMTi) in human and bovine cells (17–19), and knockdown of a histone demethylase reduces HDP gene expression in human skin keratinocytes (20), implying the benefit of demethylating DNA and histones in HDP expression. However, no study has tested a possible synergy between increased histone acetylation and decreased DNA/histone methylation in HDP gene induction.

We recently developed a luciferase reporter cell-based high throughput screening assay to identify small-molecule compounds with the ability to induce HDP gene expression by activating the gene promoter (21–23). Using such an assay, we have identified a number of HDACi, DNMTi, and HMTi that are capable of inducing HDP gene expression individually (21, 22). In this study, we explored and further confirmed for the first time a strong synergy between HDACi and DNMTi and between HDACi and HMTi in HDP induction. Our results suggest the potential of using a combination of these epigenetic compounds as a novel antibiotic-free approach to disease control and prevention in livestock animals and possibly humans as well.

## Materials and Methods

### Cell Culture Media and Chemicals

**Figure 1:**
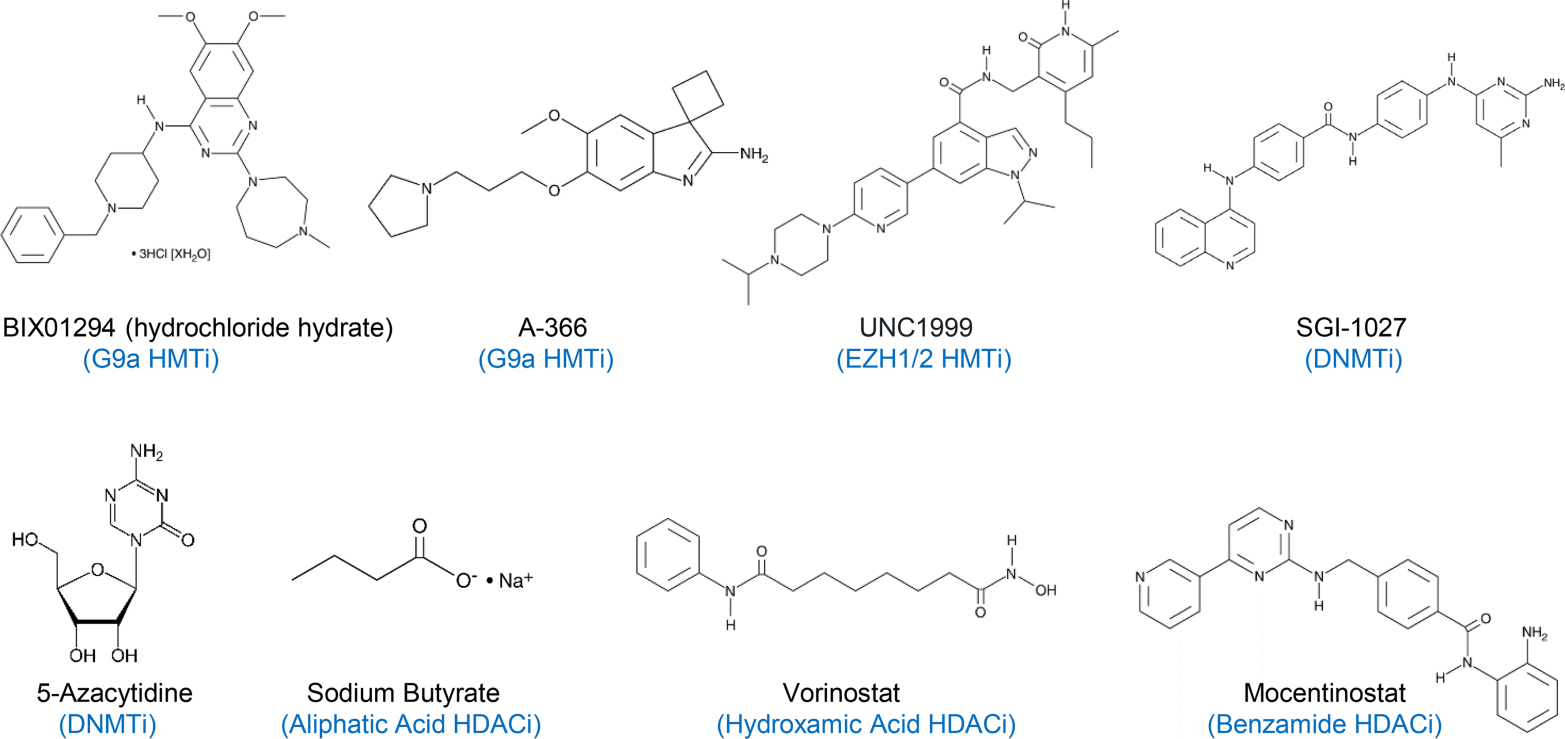
A list of histone methyltransferase inhibitors (HMTi), DNA methyltransferase inhibitors (DNMTi), and histone deacetylase inhibitors (HDACi) used in this study. BIX01294 and A-366 are G9a-specific HMTi, while UNC1999 is a specific EZH1/2 HMTi.

RPMI 1640 medium, penicillin, streptomycin, and puromycin were purchased from Hyclone (Logan, UT), while fetal bovine serum (FBS) was obtained from Atlanta Biologicals (Lawrenceville, GA). Sodium butyrate and bacterial lipopolysaccharide (LPS) from *Escherichia coli* O55:B5 was procured from MilliporeSigma (St. Louis, MO), and BIX01294, A-366, UNC1999, SGI-1027, 5-azacytidine (AZA), vorinostat, and mocetinostat (**Figure 1**) were acquired from Cayman Chemical (Ann Arbor, Michigan). Sodium butyrate and LPS were dissolved in RPMI 1640, while all other chemicals were dissolved in dimethyl sulfoxide (DMSO). In all subsequent cell culture experiments, cells treated with an equal volume of RPMI 1640 or DMSO were used as negative controls to the cells treated with individual compounds or their combinations.

### Culture and Stimulation of Macrophages

Two different chicken macrophage cell lines, HTC (24) and HD11 (25), was kindly provided by Dr. Narayan C. Rath and Dr. Hyun Lillehoj of USDA-ARS, respectively, and maintained in RPMI 1640 containing 10% heat-inactivated FBS, 100 U/mL penicillin, and 100 μg/mL streptomycin and subcultured every 3-4 days. After overnight seeding at 1.5 × 10^6^ cells/well in 6-well cell culture plates, cells were treated with different concentrations of each compound for 24 h or with the indicated concentration of each compound for various lengths of time for dose-response and time-course experiments, respectively. To study the synergy between HMTi/DNMTi and HDACi, different concentrations of an HMTi or DNMTi were added to the cells in the presence or absence of an HDACi for 24 h, followed by RNA isolation and HDP gene expression analysis as described below. HTC cells were also stimulated with butyrate, BIX01294, or their combination for 24 h, followed by 3-h stimulation with 10 ng/mL LPS and subsequent expression analysis.

### Culture and Stimulation of a Stable HTC/*AvBD9-luc* Luciferase Reporter Cell Line

A stable HTC/*AvBD9-luc* stable cell line was generated by cloning a 2.0-Kb *AvBD9* gene promoter fragment into a lentiviral luciferase reporter vector, pGreenFire1-mCMV-Puro (System Biosciences, Palo Alto, CA) and then transfected into HTC cells as we described (22). The stable cell line was maintained in RPMI 1640 containing 10% heat-inactivated FBS, 100 U/mL penicillin, 100 μg/mL streptomycin, and 0.5 µg/mL puromycin and subcultured every 3-4 days. To measure transcriptional activation of the *AvBD9* gene promoter in response to different epigenetic compounds, cells were seeded at 4 × 10^4^ cells/well in white 96-well tissue culture plates and stimulated in duplicate with different compounds individually or in combination for 24 h. An equal amount of a solvent was applied as a negative control. Luciferase activity was measured using Steady-Glo^®^ Luciferase Assay System (Promega, Madison, WI) on an L-Max II luminescence microplate reader (Molecular Devices, Sunnyvale, CA). Cell viabilities were assessed by adding alamarBlue Reagent (ThermoFisher, Waltham, MA) to cell culture 4 h before luciferase assay. Relative luciferase activity was determined for each well after normalization to the cell viability as we described (22).

### Isolation, Culture, and Stimulation of Chicken Peripheral Blood Mononuclear Cells (PBMCs)

Chicken PBMCs were isolated from EDTA-anticoagulated blood collected from 2- to 4-week-old broiler chickens through gradient centrifugation using Histopaque 1077 (MilliporeSigma) as previously described (22, 26). PBMCs were seeded at 3 × 10^7^ cells/well in 6-well cell culture plates and stimulated with different concentrations of an HMTi or DNMTi with or without 1 mM butyrate for 24 h, followed by RNA isolation and HDP gene expression analysis as described below.

### Total RNA Extraction and Gene Expression Analysis

Total RNA was extracted using RNAzol (Molecular Biology Research Center, Cincinnati, OH) according to the manufacturer’s protocol. Gene expression was performed using reverse transcription-quantitative PCR (RT-qPCR) as we described (27–29). Reverse transcription was performed with 300 ng of total RNA and Maxima^®^ First Strand cDNA Synthesis Kit (ThermoFisher Scientific, Pittsburgh, PA). The cDNA was then diluted 4-fold with RNase-free water prior to real-time PCR using QuantiTect SYBR^®^ Green PCR Kit (Qiagen, Valencia, CA) in 10-μL reactions using primers specific for HDP, barrier function, and inflammatory cytokine genes as described (27, 29, 30). PCR was run in CFX Connect Real-Time PCR System (Bio-Rad, Hercules, CA) using the following program: 95°C for 10 min, followed by 40 cycles of 94°C for 15 sec, 55°C for 20 sec, and 72°C for 30 sec. The specificity of PCR reactions was confirmed by the melting curve analysis. The glyceraldehyde-3-phosphate dehydrogenase (*GAPDH*) gene was used as the reference for data normalization. The fold changes in gene expression in the cells treated with individual compounds or in combinations were calculated relative to the cells treated with an equal volume of RPMI 1640 or DMSO using the ΔΔCt method (31).

### Statistical Analysis

Data analysis and visualization were implemented in GraphPad Prism (GraphPad Software, La Jolla, CA). Results were presented as means ± standard error of the mean (SEM) and compared among treatments using one-way analysis of variance and *post hoc* Tukey’s test. Statistical significance was considered if *P* < 0.05.

## Results

### HMTi and DNMTi Induce *AvBD9* Gene Expression in Concentration- and Time-Dependent Manners

**Figure 2:**
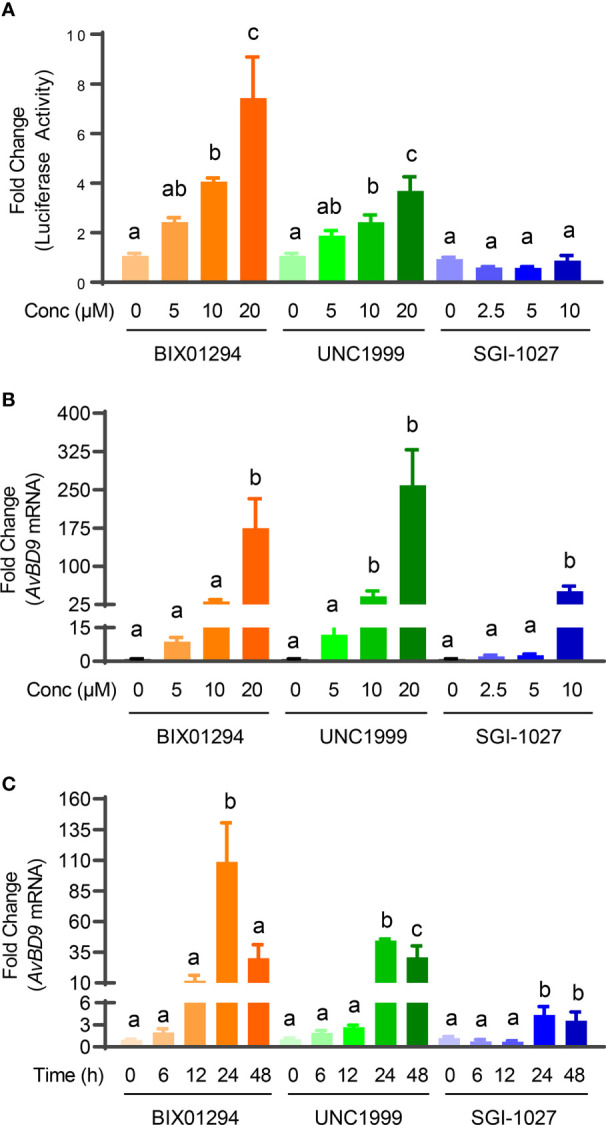
Concentration- and time-dependent induction of *AvBD9* mRNA expression by HMTi or DNMTi. **(A)** Fold changes in relative luciferase activity in stable chicken HTC/*AvBD9-luc* luciferase reporter cells in response to indicated concentrations (μM) of each compound for 24 h. **(B)** Fold changes in the *AvBD9* mRNA expression level in chicken HTC cells stimulated with indicated concentrations of each compound for 24 h. **(C)** Fold changes in the *AvBD9* mRNA expression level in chicken HTC cells stimulated with 10 µM of each compound for indicated lengths of time. *AvBD9* mRNA expression was analyzed using RT-qPCR. Results are shown as means ± SEM of three independent experiments. The bars not sharing a common superscript are considered significantly different (*P* < 0.05) based on one-way analysis of variance and *post hoc* Tukey’s test.

We previously identified a number of epigenetic compounds including HDACi, HMTi, and DNMTi that are capable of inducing HDP gene expression using luciferase reporter cell lines driven by HDP gene promoters (21, 22). HDACi are well-known HDP inducers (10, 16). To further evaluate whether newly-identified HMTi (BIX01294 and UNC1999) and a DNMTi (SGI-1027) can induce HDP gene expression in both concentration- and dose-dependent manners, chicken HTC/*AvBD9-luc* reporter cell line was initially treated with different concentrations of each compound for 24 h. BIX01294 and UNC1999 showed an obvious concentration-dependent activation of the *AvBD9* gene promoter in the reporter cell line as indicated by increases in relative luciferase activity, but SGI-1027 was a weak activator (**Figure 2A**). BIX01294 and UNC1999 were further confirmed to induce *AvBD9* mRNA expression in chicken HTC macrophage cell line in a concentration-dependent fashion peaking with a 175- and 259- fold increase in response to 20 µM of each compound, respectively, while 10 µM SGI-1027 gave a 51-fold induction (**Figure 2B**). Because it triggered a robust *AvBD9* mRNA induction with each compound, 10 µM was used in subsequent time-course experiments. All three compounds also showed a time-dependent induction of *AvBD9* mRNA expression in HTC cells peaking at 24 h (**Figure 2C**). Therefore, cells were treated for 24 h for all downstream analyses of HDP gene expression.

### HMTi or DNMTi Synergizes With Butyrate to Induce HDP Gene Expression

**Figure 3:**
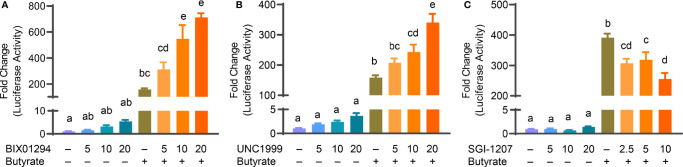
Synergistic induction of luciferase activity in HTC/*AvBD9-luc* cells between butyrate and an HMTi or a DNMTi. Chicken HTC/*AvBD9-luc* luciferase reporter cells were stimulated in duplicate with indicated concentrations (μM) of BIX01294 **(A)**, UNC1999 **(B)**, or SGI-1027 **(C)** individually or in combination with 2 mM butyrate for 24 h, followed by luciferase assay for the *AvBD9* gene promoter activity. Results are shown as means ± SEM of three independent experiments. The bars not sharing a common superscript are considered significantly different (*P* < 0.05) based on one-way analysis of variance and *post hoc* Tukey’s test.

**Figure 4:**
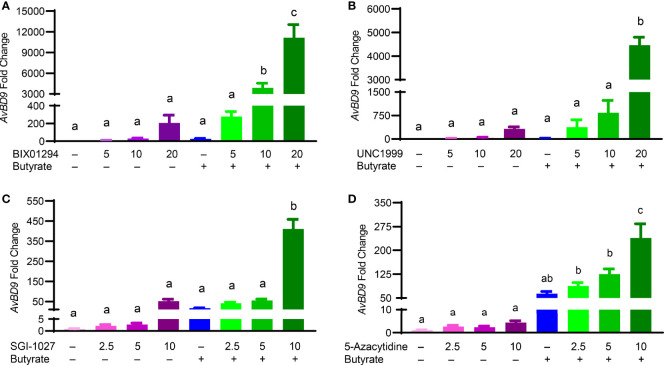
Synergistic induction of the *AvBD9* mRNA expression in HTC cells between HMTi and butyrate or between DNMTi and butyrate. Chicken HTC cells were stimulated in duplicate with indicated concentrations (μM) of BIX01294 **(A)**, UNC1999 **(B)**, SGI-1027 **(C)**, or 5-azacytidine **(D)** individually or in combination with 2 mM butyrate for 24 h, followed by RT-qPCR analysis of *AvBD9* mRNA expression. Results are shown as means ± SEM of three independent experiments. The bars not sharing a common superscript are considered significantly different (*P* < 0.05) based on one-way analysis of variance and *post hoc* Tukey’s test.

Butyrate, an HDACi, is well-known to induce HDP gene expression in different animal species including humans and chickens (26, 32). Sodium butyrate at 2 mM gives optimal HDP gene induction in chicken HTC and HD11 cells and further synergizes strongly with several other HDP-inducing compounds such as forskolin (27, 30), sugars (28), and cyclooxygenase-2 inhibitors (33). To explore a possible synergy between butyrate and an HMTi or DNMTi, we treated HTC/*AvBD9-luc* reporter cells with different concentrations of BIX01294, UNC1999, or SGI-1027 in the presence or absence of 2 mM butyrate for 24 h. A marked dose-dependent increase in the synergy in luciferase activity was observed between butyrate and BIX01294 (**Figure 3A**) as well as between butyrate and UNC1999 (**Figure 3B**). However, no synergy between butyrate and SGI-1027 was observed in the luciferase reporter cell line (**Figure 3C**). HTC cells were used to confirm whether the synergy occurs between butyrate and an HMTi or DNMTi to induce *AvBD9* mRNA expression. A concentration-dependent increase in *AvBD9* mRNA expression was indeed observed between butyrate and BIX01294, UNC1999, or SGI-1027. For example, 20 µM BIX01294 and butyrate individually induced *AvBD9* expression by 207- and 27-fold, respectively; however, a combination of 20 µM BIX01294 and butyrate gave a remarkable 11,165-fold increase in *AvBD9* expression, which is an additional 54-fold increase over 20 µM BIX01294 alone (**Figure 4A**). Similarly, 20 µM UNC1999 enhanced *AvBD9* expression by 324-fold, but a UNC1999/butyrate combination gave a 4,470-fold increase, which reflected an additional 14-fold increase over UNC1999 alone (**Figure 4B**). SGI-1027 at 10 µM augmented *AvBD9* expression by 51-fold, but further induced *AvBD9* by 411-fold when combined with butyrate, which is an additional 8-fold increase over 10 µM SGI-1027 alone (**Figure 4C**). AZA, another well-known DNMTi (34), was also examined and confirmed its synergy with butyrate in a concentration-dependent manner (**Figure 4D**). It is noted that the *AvBD9*-inducing synergy between butyrate and an HMTi tended to be stronger than between butyrate and a DNMTi.

**Figure 5:**
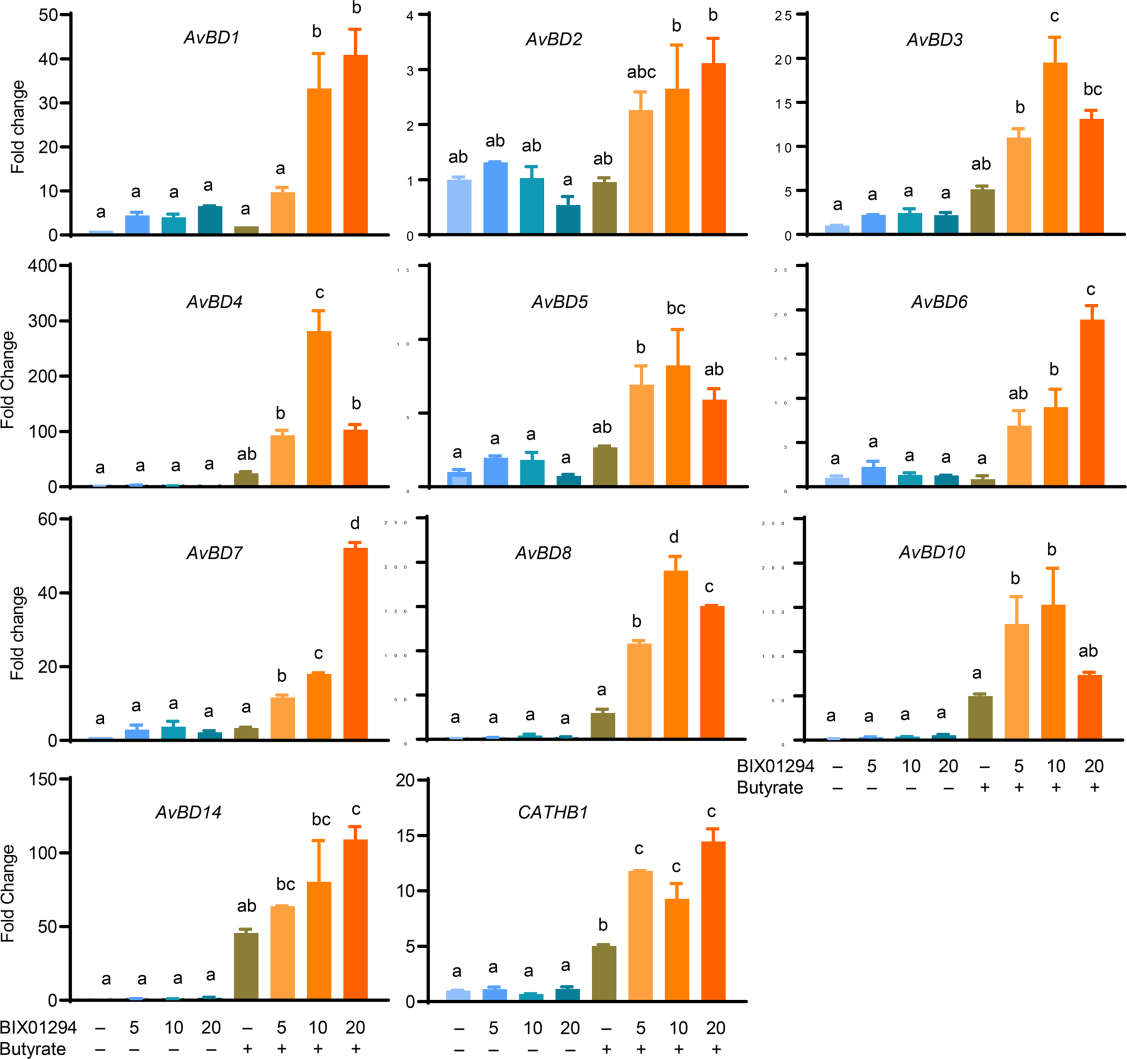
Synergistic induction of multiple HDP mRNA expression in HTC cells between BIX01294 and butyrate. Chicken HTC cells were stimulated in duplicate with indicated concentrations (μM) of BIX01294 with or without 2 mM butyrate for 24 h, followed by RT-qPCR analysis of different chicken HDP genes. Results are shown as means ± SEM of two independent experiments. The treatments not sharing a common superscript are considered significantly different (*P* < 0.05) based on one-way analysis of variance and *post hoc* Tukey’s test.

Moreover, among those HDP genes that are detectable in HTC cells, all were synergistically induced in response to both BIX01294 and butyrate, although different magnitudes of induction were observed with different HDP genes. For example, *AvBD2* was only upregulated by approximately 2- to 3-fold by a combination of butyrate and BIX01294, while *AvBD4*, *AvBD8*, *AvBD10*, and *AvBD14* were induced by greater than 100-fold in response to butyrate and at least one concentration of BIX01294, with the remaining HDP genes showing an intermediate response (**Figure 5**).

**Figure 6:**
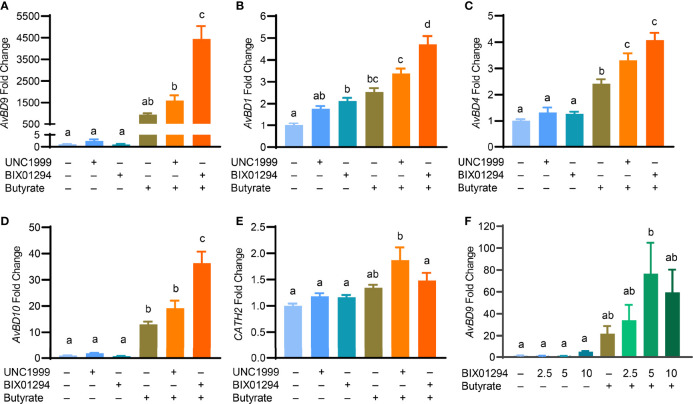
Synergistic induction of the HDP mRNA expression in HD11 cells and peripheral blood mononuclear cells (PBMCs) between butyrate and an HMTi. Chicken HD11 macropahges were stimulated in duplicate with 2 mM butyrate with or without either 5 μM BIX01294 or 5 μM UNC1999 for 24 h, followed by RT-qPCR analysis of the gene expression levels of *AvBD9* **(A)**, *AvBD1* **(B)**, *AvBD4* **(C)**, *AvBD10* **(D)**, and *CATH2* **(E)**. **(F)** Chicken PBMCs were stimulated in duplicate with indicated concentrations (μM) of BIX01294 in the presence or absence of 1 mM butyrate for 24 h, followed by RT-qPCR of *AvBD9* expression. Results are shown as means ± SEM of 2-3 independent experiments. The treatments not sharing a common superscript are considered significantly different (*P* < 0.05) based on one-way analysis of variance and *post hoc* Tukey’s test.

To confirm whether the HDP-inducing synergy between butyrate and an HMTi is preserved in a different cell line, chicken HD11 macrophages were stimulated with butyrate in the presence or absence of BIX01294 or UNC1999 for 24 h. Consistent with the results in HTC cells, a drastic synergy in *AvBD9* induction was observed between butyrate and BIX01294 and between butyrate and UNC1999 (**Figure 6A**). Similarly, *AvBD1* (**Figure 6B**), *AvBD4* (**Figure 6C**), and *AvBD10* (**Figure 6D**) were also synergistically induced albeit at lower magnitudes. On the other hand, *CATH2* mRNA was minimally regulated by individual compounds or their combinations in HD11 cells (**Figure 6E**). To further verify a possible HDP-inducing synergy between butyrate and BIX01294 in primary cells, chicken PBMCs were isolated and treated with BIX01294 in the presence or absence of butyrate for 24 h. BIX01294 alone induced *AvBD9* in a dose-dependent manner, and the BIX01294/butyrate combination showed an obvious synergy (**Figure 6F**).

### HMTi or DNMTi Synergizes With HDACi to Induce HDP Gene Expression

**Figure 7:**
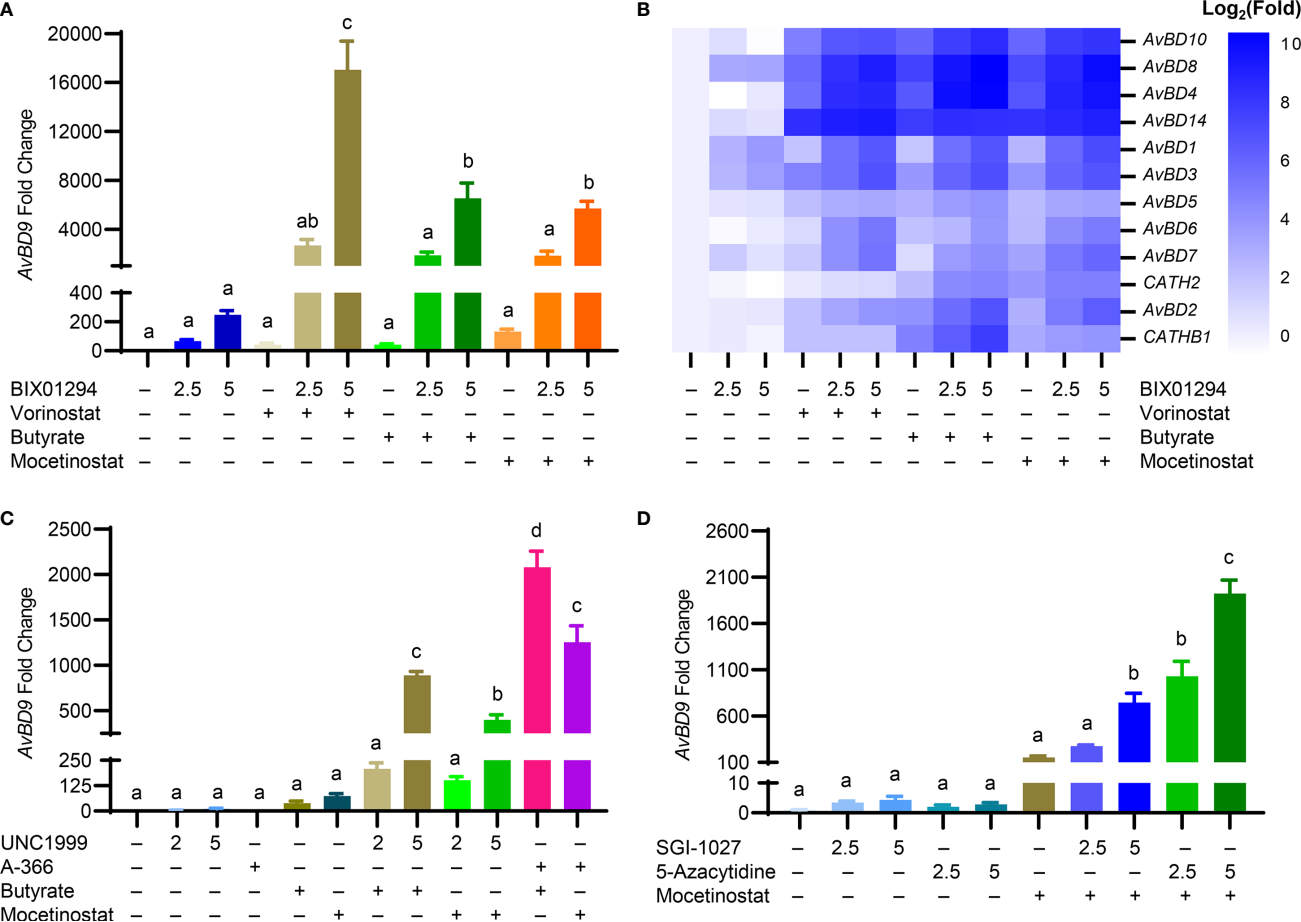
Synergistic induction of chicken HDP mRNA expression in HTC cells between HMTi and HDACi or between DNMTi and HDACi. Chicken HTC cells were stimulated in duplicate with an HMTi (2.5 or 5 μM BIX01294) in the presence or absence of one of three HDACi (5 μM SAHA, 2 mM butyrate, or 2.5 μM mocetinostat) for 24 h, followed by RT-qPCR analysis of the expressions of *AvBD9* **(A)** and other chicken HDP genes **(B)**. **(C)** Chicken HTC cells were stimulated in duplicate with an HMTi (2 or 5 μM UNC1999 or 5 μM A-366) individually or in combination with an HDACi (2 mM butyrate or 2.5 μM mocetinostat) for 24 h, followed by RT-qPCR analysis of the expressions of *AvBD9* gene. **(D)** Chicken HTC cells were stimulated in duplicate with a DNMTi (2.5 or 5 μM SGI-1027 or 5-azacytidine) individually or in combination with a HDACi (2 mM butyrate or 2.5 μM mocetinostat) for 24 h, followed by RT-qPCR analysis of *AvBD9* expression. Results are shown as means ± SEM of three independent experiments. The bars not sharing a common superscript are considered significantly different (*P* < 0.05) based on one-way analysis of variance and *post hoc* Tukey’s test.

To further examine whether other structurally different HDACi also synergize with BIX01294 in *AvBD9* induction, chicken HTC cells were treated with different concentrations of BIX01294 with or without an HDACi (vorinostat, butyrate, and mocetinostat) (**Figure 1**). A dramatic synergy in *AvBD9* induction was observed between BIX01294 and any of three HDACi. For example, 5 μM BIX01294 in combination with vorinostat, butyrate, and mocetinostat gave an additional 69-, 26-, and 23-fold increase in *AvBD9* gene expression over BIX01294 alone, respectively (**Figure 7A**). In addition to *AvBD9*, most other HDP genes were also synergistically induced in response to a combination of BIX01294 and any of the three HDACi (**Figure 7B**). To study whether other HMTi also synergize with HDACi in HDP induction, HTC cells were treated with UNC1999 or A-366 (**Figure 1**) in the presence or absence of butyrate or mocetinostat. Indeed, a marked synergy was seen between any two-compound combination of an HMTi (UNC1999 or A-366) and an HDACi (butyrate or mocetinostat) (**Figure 7C**).

Butyrate was shown to synergize in HDP gene induction with SGI-1027 and AZA (**Figures 4C, D**). We sought to further examine a possible synergy between a DNMTi and a structurally different HDACi. Unsurprisingly, a drastic synergy was observed between mocetinostat and either DNMTi. For example, 5 μM SGI-1027, 5 μM AZA, and 2.5 μM mocetinostat induced *AvBD9* gene expression by 4-, 3-, and 152-fold, respectively, while a combination of 5 μM SGI-1027/mocetinostat and 5 μM AZA/mocetinostat gave 747- and 1,925-fold increases in *AvBD9* expression (**Figure 7D**).

### HMTi and Butyrate Cooperate to Regulate the Genes Involved in Barrier Function and Inflammation

**Figure 8:**
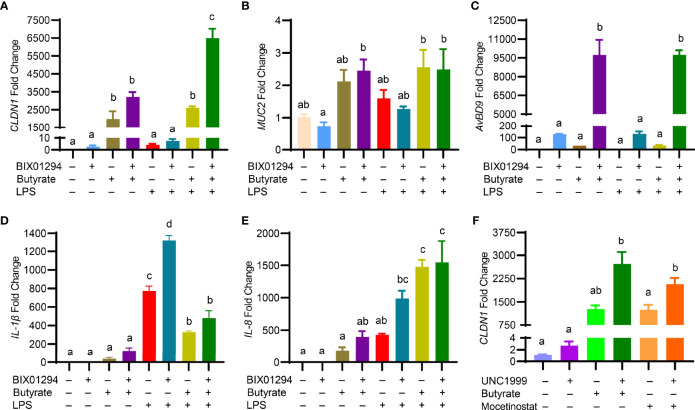
Regulation of the genes involved in barrier function, host defense, and inflammation in HTC cells by HMTi and HDACi. Chicken HTC cells were stimulated in duplicate with 5 μM BIX01294 and 2 mM butyrate individually or in combination for 24 h, followed by 3-h stimulation with 10 ng/ml lipopolysaccharide (LPS) for another 3 h. RT-qPCR was performed to analyzed the expressions of *CLND1* **(A)** *, MUC2* **(B)** *, AvBD9* **(C)**, *IL-1β* **(D)**, and *IL-8* **(E)**. **(F)** Chicken HTC cells were stimulated in duplicate with 5 μM UNC1999 or in combination with 2 mM butyrate or 2.5 μM mocetinostat for 24 h, followed by RT-qPCR analysis of *CLND1* expression. Results are shown as means ± SEM of three independent experiments. The bars not sharing a common superscript are considered significantly different (*P* < 0.05) based on one-way analysis of variance and *post hoc* Tukey’s test.

Butyrate is known to enhance mucosal barrier function by inducing the genes for mucin 2 (*MUC2*) and tight junction proteins such as claudin 1 (*CLDN1*) and tight junction protein 1 (*TJP1*) while suppressing inflammation (35). To examine the impact of the butyrate/BIX01294 combination on barrier function and inflammation, HTC cells were treated with butyrate and BIX01294 individually or in combination for 24 h, followed by a 3-h stimulation with LPS. To our surprise, although BIX01294 had a minimum activity in increasing *CLDN1* expression, a marked synergy was observed in response to a combination of BIX01294 and butyrate, and the synergy was further enhanced in response to subsequent LPS treatment (**Figure 8A**). However, *MUC2* (**Figure 8B**) and *TJP1* (data not shown) were minimally affected in HTC cells by the compounds or their combination with or without LPS, while it is desirable that LPS had no impact on the *AvBD9*-inducing synergy (**Figure 8C**).

Interestingly, although both butyrate and BIX01294 had no or weak activity in inducing gene expressions of inflammatory cytokines *IL-1β* (**Figure 8D**) and IL-8 (**Figure 8E**), the combination tended to have a synergistic effect on triggering the synthesis of both cytokines. While butyrate suppressed LPS-induced *IL-1β* expression, BIX01294 had an opposite effect, and a combination of butyrate and BIX01294 led to a significant reduction in *IL-1β* expression (**Figure 8D**). Both butyrate and BIX01294 potentiated LPS-induced *IL-8* expression individually, and the IL-8 expression level remained elevated in response to the combination (**Figure 8E**). It is important to note that *CLDN1* was also synergistically induced by butyrate and UNC1999 or by mocetinostat and UNC1999 (**Figure 8F**), suggesting that a combination of HDACi and HMTi is synergistic in augmenting *CLDN1* gene expression; however, *MUC2* and *TJP1* genes were minimally regulated by HDACi and HMTi (data not shown).

## Discussion

Enhancing the synthesis of endogenous HDPs has been shown to be effective against microbial infections and, therefore, is actively explored as a novel antibiotic-free, host-directed approach to antimicrobial therapy (9–11). We recently identified a number of HDACi, HMTi, and DNMTi with the ability to induce HDP gene expression from several high throughput screening efforts (21, 22) (and unpublished results). In this study, we confirmed for the first time a strong synergy between three structurally distinct HDACi (butyrate, vorinostat, or mocetinostat) and three HMTi (BIX01294, A-366, or UNC1999) or two DNMTi (SGI-1027 or AZA) (**Figure 1**) in HDP gene induction in two chicken HTC macrophage cell lines and PBMCs. Additionally, most HDP genes appear to be upregulated by the two-compound combinations, suggesting the potential of these epigenetic compounds for disease control and prevention.

Gene transcription is controlled by not only the DNA sequence, but also the accessibility of a gene promoter, while the latter is dictated by the modification status of histones and DNA in the region (12, 13). Among major epigenetic modifications of histones are acetylation and methylation (36, 37). The lysine residues in the amino-terminal tail of histones can be acetylated by HAT and deacetylated by HDAC. Acetylation of the lysine residues reduces the positive charge of the histones and subsequent binding of the histone tails with negatively charged DNA, thereby leading to a more relaxed and accessible chromatin, which facilitates the binding of transcription factors and transcriptional machinery for subsequent gene transcription (36, 37). Additionally, acetylated lysines also recruit bromodomain-containing transcription factors to the gene promoter for active transcription (38, 39). On the other hand, histone deacetylation leads to an opposite outcome with a more condensed chromatin and decreased gene transcription. Among four classes of HDAC, HDAC1 is primarily responsible for the constitutive expression of human β-defensin 1 (*DEFB1*) in A549 lung epithelial cell line (40). HDACi function to maintain the acetylation status of chromatin and thereby facilitate gene transcription (41). A number of structurally different HDACi such as butyrate and entinostat have been found to have the HDP-inducing activity (10, 16).

Histones can also be methylated on the lysine or arginine residues by HMT or demethylated by histone demethylases (14). Histone methylation can lead to either an increase or decrease in gene transcription, depending largely upon the site of methylation. For example, methylation of lysine 4 of histone 3 (H3K4) leads to transcriptional activation, while methylation of H3K9, H3K20, and H3K27 signals transcriptional suppression (14). G9a, also known as euchromatic histone-lysine N-methyltransferase 2 (EHMT2), catalyzes methylation of H3K9 and H3K27 leading to gene silencing, while enhancer of zeste homolog 1/2 (EZH1/2) are another class of HMT that primarily catalyze methylation of H3K27 to suppress gene transcription (42). Trimethylation of H3K27 has been found to be negatively associated with HDP synthesis in human skin keratinocytes (20). Therefore, it is possible that inhibitors of G9a or EZH1/2 HMT could potentially lead to enhanced HDP synthesis.

Similarly, DNA can also be methylated at the C5 position of CpG dinucleotides by DNMT, which often leads to gene silencing by impairing the binding of transcription activators or facilitating the binding of transcription suppressors with high affinity for methylated CpG (14). Hypermethylation of the gene promoters has been found to be associated with reduced expression of two human β-defensin genes (*DEFB1* and *DEFB4*) in prostate cancer cells (43) and oral carcinoma cells (17), respectively. Conversely, treatment of human oral carcinoma cells (17), vaginal keratinocyte cells (44), or bovine mammary epithelial cells (19) with a DNMTi induces HDP gene expression.

In this study, three structurally distinct HDACi (butyrate, vorinostat, and mocetinostat), three HMTi (BIX01294, A-366, and UNC1999), and two DNMTi (AZA and SGI-1027) were used to evaluate their synergy. Consistent with earlier reports, these epigenetic compounds are capable of inducing HDP gene expression in chicken HTC macrophages, suggesting these genes can be regulated by histone acetylation and DNA/histone methylation. Importantly, we observed a dramatic synergy in induction of most HDP genes in response to a combination of HDACi and HMTi or a combination of HDACi and DNMTi, implying therapeutic and prophylactic potential for these compounds against infectious diseases. Follow-up animal studies will be needed to confirm the *in vivo* efficacy of these compounds in disease control and prevention.

It is noted that butyrate, vorinostat (also known as suberoylanilide hydroxamic acid or SAHA), and mocetinostat are all pan-HDACi capable of inhibiting multiple classes of HDAC (45). It is important to study relative HDP-inducing potency of those HDAC inhibitors that are specific for a certain class of HDAC. Given the major involvement of HDAC1 in human *DEFB1* expression, HDACi with a strong ability to suppress HDAC1 may be potent in HDP gene induction (40). However, further investigations are warranted on whether such a mechanism is conserved among individual HDP genes across different animal species. While BIX01294 and A-366 are G9a-specific HMTi responsible mainly for suppression of H3K9 methylation (46), UNC1999 is an EZH1/EZH2-specific inhibitor that specifically suppresses H3K27 methylation (47). It is expected all HMTi that can alleviate repressive methylation marks of histones (e.g., H3K9 and H3K27) will induce HDP genes, while those HMTi that suppress transcriptionally active methylation marks (e.g., H3K4) will not.

The mechanism of the synergy in HDP gene induction between HDACi and HMTi or between HDACi and DNMTi remains to be studied. However, the cross-talk is well known between epigenetic modifications in controlling gene transcription. For example, methylated CpG-binding protein 2 (MeCP2) has a high affinity for methylated DNA, which in turn recruits HDAC leading to histone deacetylation, chromatin compaction, and gene silencing (48). Additionally, MeCP2 has been found to associate with HMT, causing H3K9 methylation leading to gene suppression (49). It is, therefore, plausible that HDACi and DNMTi/HMTi could synergize chromatin relaxation and gene transcription. To our surprise, combination therapies involving different classes of epigenetic modifiers are being explored for treatment of different types of cancers (50, 51). It remains unknown whether there is a link between induced HDP expression and enhanced apoptosis of tumor cells in response to combinations of epigenetic modifiers. Nevertheless, our discovery of the synergistic action in HDP gene induction between HDACi and DNMTi/HMTi may lead to the development of a novel host-directed antimicrobial therapy against infectious diseases.

In addition to HDPs, CLDN1, the most studied transmembrane protein involved in the assembly of tight junctions (52), is also synergistically induced by HDACi and HMTi. However, a different tight junction protein, TJP1 and the predominant mucous component in the digestive tract, MUC2 (53), are minimally affected. While it is capable of suppressing LPS-induced *IL-1β* expression, a combination of butyrate and BIX01294 appears to further potentiate LPS-induced *IL-8* expression. Therefore, the significance of HDACi, HMTi, and DNMTi in regulating innate immunity, barrier function, and inflammatory response in live animals as well as their *in vivo* safety and efficacy in disease control and prevention warrants further investigation. Additionally, it is important to evaluate the impact of these epigenetic compounds on regulating other genes and biological processes.

## Conclusions

In this study, we explored and revealed for the first time a marked synergy between HDACi and HMTi and between HDACi and DNMTi in the induction of multiple HDP genes in two different macrophage cell lines. We further verified the HDP-inducing synergy between butyrate, an HDACi, and BIX01294, an HMTi, in PBMCs. Additionally, we revealed that several important genes involved in barrier function and inflammatory response are differentially regulated by HDACi and HMTi. We conclude that epigenetics play an important role in regulating HDP gene expression. Strategies to increase histone acetylation while reducing DNA or histone methylation could synergistically enhance HDP gene expression, and therefore, have potential for disease control and prevention.

## Data Availability Statement

The original contributions presented in the study are included in the article/supplementary material. Further inquiries can be directed to the corresponding author.

## Author Contributions

MW, HL, and WL conducted the experiments. MW, HL, WL, and GZ performed data analysis. HL and SK drafted the manuscript. GZ conceived the study and revised the manuscript. All authors contributed to the article and approved the submitted version.

## Funding

This research was funded by the USDA National Institute of Food and Agriculture (grant no. 2018-68003-27462, 2020-67016-31619, and 2022-67016-37208), Oklahoma Center for the Advancement of Science and Technology (grant no. AR19-027), the Ralph F. and Leila W. Boulware Endowment Fund, and Oklahoma Agricultural Experiment Station Project H-3112. MAW was supported by a USDA Predoctoral Fellowship (grant no. 2021-67034-35184).

## Conflict of Interest

A provisional patent describing the synergy between histone deacetylase inhibitors and DNA/histone methyltransferase inhibitors was recently filed with the U.S. Patent and Trademark Office with GZ and MW being co-inventors.

The remaining authors declare that the research was conducted in the absence of any commercial or financial relationships that could be construed as a potential conflict of interest.

## Publisher’s Note

All claims expressed in this article are solely those of the authors and do not necessarily represent those of their affiliated organizations, or those of the publisher, the editors and the reviewers. Any product that may be evaluated in this article, or claim that may be made by its manufacturer, is not guaranteed or endorsed by the publisher.

## Abbreviations

AvBD9, avian β-defensin 9; AZA, 5-azacytidine; CLDN1, claudin 1; DEFB1, human β-defensin 1; DMSO, dimethyl sulfoxide; DNMT, DNA methyltransferase; DNMTi, DNMT inhibitor; GAPDH, glyceraldehyde-3-phosphate dehydrogenase; HDAC, histone deacetylase; HDACi, HDAC inhibitor; HDPs, host defense peptides; HMT, histone methyltransferase inhibitor; HMT, HMT inhibitor; MUC2, mucin 2; PBMCs, peripheral blood mononuclear cells; RT-qPCR, reverse transcriptase-quantitative PCR; SEM, standard error of the mean; TJP1, tight junction protein 1.
